# A role for IFT88/the primary cilium in the inflammatory response to interleukin-1

**DOI:** 10.1186/2046-2530-1-S1-P60

**Published:** 2012-11-16

**Authors:** AKT Wann, MM Knight

**Affiliations:** 1 Queen Mary, University of London, UK

## 

The assembly and maintenance of the primary cilium is managed, in part, by intraflagellar transport (IFT). Many diseases comprise an inflammatory component, often associated with elevated cytokine concentrations. Here we test the hypothesis that the primary cilium has a role in inflammatory responses during pathology. This study used murine chondrocytes with a hypomorphic mutation of *Tg737* (encoding IFT88) that abolishes cilia growth. Cells were treated with the pro-inflammatory cytokine IL-1β and the release of prostaglandin E2 (PGE2) and nitric oxide was quantified using ELISA and the Greiss assay respectively. Cilia were labelled with anti-acetylated tubulin, imaged using confocal microscopy and length and prevalence quantified. Exposure of WT chondrocytes to IL-1β resulted in increased PGE2 and nitric oxide release. For *Tg737* cells, IL-1-induced PGE2 release was significantly attenuated and the up-regulation of nitric oxide abolished. IL-1 treatment, including different doses and subtypes, induced a consistent ~50% increase in cilia length. Increases were also observed in NIH3T3 cells. Inhibition of PKA abolished IL-1-induced elongation. Studies also indicate roles for PKC and the MEK-ERK pathways, already known to be downstream of IL-1 effects, in IL-1-induced cilia elongation. We believe this is exciting preliminary evidence for a role for the primary cilium in cytokine-induced inflammation and suggest that the primary cilium may have a role in inflammatory pathologies. Due to the localised, specific nature of much of the cilia proteome, an understanding of the role of IFT and the cilia structure regulation in inflammation will help in the hunt for novel therapeutic strategies.

